# Low-Energy Desalination Techniques, Development of Capacitive Deionization Systems, and Utilization of Activated Carbon

**DOI:** 10.3390/ma17205130

**Published:** 2024-10-21

**Authors:** Gaber A. Elawadi

**Affiliations:** Department of Mechanical Engineering, College of Engineering and Computer Sciences, Jazan University, 114 Almarefah Rd., Jazan 45142, Saudi Arabia; gaberelawdi1964@gmail.com

**Keywords:** activated carbon, desalination, CDI, electrode, volt, TDS, flow rate

## Abstract

Water desalination technology has emerged as a critical area of research, particularly with the advent of more cost-effective alternatives to conventional methods, such as reverse osmosis and thermal evaporation. Given the vital importance of water for life and the scarcity of potable water for agriculture and livestock—especially in the Kingdom of Saudi Arabia—the capacitive deionization (CDI) method for removing salt from water has been highlighted as the most economical choice compared to other techniques. CDI applies a voltage difference across two porous electrodes to extract salt ions from saline water. This study will investigate water desalination using CDI, utilizing a compact DC power source under 5 volts and a standard current of 2 amperes. We will convert waste materials like sunflower seeds, peanut shells, and rice husks into activated carbon through carbonization and chemical activation to improve its pore structure. Critical parameters for desalination, including voltage, flow rate, and total dissolved solids (TDS) concentration, have been established. The initial TDS levels are set at 2000, 1500, 1000, and 500 ppm, with flow rates of 38.2, 16.8, and 9.5 mL/min across the different voltage settings of 2.5, 2, and 1.5 volts, applicable to both direct and inverse desalination methods. The efficiency at TDS concentrations of 2000, 1500, and 1000 ppm remains between 18% and 20% for up to 8 min. Our results indicate that the desalination process operates effectively at a TDS level of 750 ppm, achieving a maximum efficiency of 45% at a flow rate of 9.5 mL/min. At voltages of 2.5 V, 2 V, and 1.5 V, efficiencies at 3 min are attained with a constant flow rate of 9.5 mL/min and a TDS of 500 ppm, with the maximum desalination efficiency reaching 56%.

## 1. Introduction

To achieve the objective of supplying everyone with safe and affordable drinking water, water treatment technology must advance through innovation. Over 97% of the water resources on Earth are saltwater, mainly seawater, making desalination an appealing alternative. Water is everywhere, along with salty lakes, estuaries, mangroves, and marshes. The total dissolved solids (TDS) concentration is a criterion that can be used to classify salt water. Freshwater is defined as having a TDS concentration of 500 ppm or less, salty water is defined as having a TDS concentration of 500 ppm to 15,000 ppm, and seawater is defined as having a TDS concentration of more than 15,000 ppm [1,2].

A typical CDI cell comprises two porous electrodes connected by a spacer, which allows water to flow between them. CDI gathers ionized species on porous electrodes to operate [1]. An applied voltage during operation causes the adsorption of ions on the electrodes, which results in the production of freshwater. A few operational parameters that impact conventional CDI procedures are the flow rate, flow delivery, applied voltage, and ion concentration. Various newly investigated materials, including carbon fiber cloth (ACC), graphene aerogels, carbon nanotubes, and graphene powders, including surface-modified variants, are used to manufacture CDI electrodes. Researchers have also developed several models to anticipate and enhance CDI processes. In other cases, high-resolution modeling has even been employed to investigate the relationships between absorption, laminar flow, and diffusion in flow-between or flow-through CDI cells. Capacitive deionization (CDI) has grown in acceptance as a dependable, affordable, and energy-efficient method for desalinating water with a low-to-moderate salt content [3,4,5,6].

The capacitive deionization (CDI) water treatment system removes ions from water through an electrochemical process [7]. CDI is a type of desalination technology that is gaining popularity since it uses less energy and is more affordable than other methods. In CDI, an electrical potential is applied using two porous electrodes that are separated from one another by a spacer. The electrical double layer at the electrode surface attracts and stores ions from the water that flows through the electrodes. Deionized water is created when the number of ions in the water decreases as it goes by the electrodes. Compared to other desalination methods, CDI provides several benefits, such as low energy consumption, low operating costs, and the ability to filter a range of ions from water [8]. The scalability of CDI makes it suitable for both small- and large-scale water treatment applications. Since CDI can remove cations and anions from water, it is an efficient way to remove contaminants such as salts, heavy metals, and organic materials. Additionally, eliminating specific ions from water via CDI, including nitrates, fluoride, and arsenic, is possible [9,10].

Since salt ions are the most minor component in water, CDI is an efficient method for purifying water with a salt concentration below 10 g/L. Various techniques are used to remove the water component of the salt solution. Energy can be recovered by charging a nearby cell participating in the ion electrosorption process with the energy released during electrode regeneration (also called electrode discharge or ion release). The CDI cycle begins with a charging stage (ion electrosorption) that purifies the water. Further in the paper, we will explore this juncture more deeply. This procedure traps ions between two porous carbon electrodes [8,9]. Desorbed ions are released from the electrodes in the next step, resulting in electrode regeneration). Capacitive deionization’s fundamental mechanism is depicted schematically. Making progress in water treatment technology is essential in providing clean, dependable drinking water. With over 97% of the planet’s water sourced from the ocean, desalination has emerged as an attractive solution. This method could be utilized in ocean water, freshwater lakes, estuarine water, mangrove ecosystems, and wetland water [11,12,13,14].

In the CDI process, a cation-exchange membrane is placed in front of the cathode, while an anion-exchange membrane is placed in front of the anode. Historically, capacitive deionization has received less attention than investigations on carbon porous electrodes for capacitive energy storage systems. However, the use of carbon porous electrodes for water desalination has been known since the 1960s, referred to as “electrochemical demineralization” or “electrosorb process for desalting water”. Only recently has academic interest and the commercialization of CDI technologies increased [4,15,16].

When an electrical voltage difference is applied, pairs of porous carbon electrodes, positioned opposite each other, store ions. These electrodes can be stacked together in multiple pairs. The ions are extracted from the water, passing through a “spacer channel” between the two electrodes, and trapped in the carbon material’s pores. This process involves forming electrical double-layered structures (EDLs) within the intraparticle pores [17,18].

Compared to other desalination techniques, CDI offers several advantages, including low energy consumption, cost-effective operation, and the ability to remove various ions from water. CDI is also scalable, making it suitable for both small- and large-scale water treatment applications [19]. CDI shows excellent promise for desalination and water treatment, with potential applications in treating brackish water, seawater, and industrial effluent while effectively removing salt [20].

Membrane capacitive deionization (MCDI) is a water desalination method that uses two opposing porous electrodes to create an electric current. As water passes through a channel between the electrodes, ions are extracted and stored within the electrodes. Ion-exchange membranes are positioned in front of the electrodes to help flow counterions from the channel into the electrode while keeping coions inside the electrode structure [21,22].

MCDI has been developed as part of an enhanced theory to consider the electrostatic forces and double layers (EDLs) formed within the porous carbon particles of the electrodes. Additionally, our theory considers the role of the inter-particle pore space as a pathway for ion transport. By preventing the coions from leaving the electrode region in MCDI, the inter-particle porosity becomes a reservoir for salt storage [23]. This improvement in total salt capacity enhances the effectiveness of the porous electrode in MCDI. One of the advantages of MCDI is its ability to reverse the voltage during ion desorption, also known as ion release. This feature allows for increased capacity and faster salt uptake in the subsequent cycle by depleting the inter-particle porosity of counter ions. In this study, we assess the adsorption and desorption cycles of MCDI, both theoretically and experimentally, for desorption at zero voltage and with reversed voltage [24,25,26].

## 2. Materials and Methods

Capacitive deionization (CDI) is a promising water treatment technology based on an electrochemical process for eliminating ions from water. This method is classified as a subtype of desalination technology. It has been gaining traction recently due to its reduced energy consumption and cost-effectiveness compared to other desalination techniques. The CDI approach applies an electrical potential across two porous electrodes separated by a spacer. Upon water flow through the electrodes, the ions in the water are drawn to the electrodes and deposited in the electrical double layer formed at the electrode surface. The concentration of ions in the water is reduced as they pass through the electrodes, resulting in deionized water [27].

This work focuses on designing, developing, and producing CDI units and synthesizing activated carbon sheets. Evaluating its efficiency in different scenarios is crucial in exploring the potential of capacitive deionization (CDI) for water desalination. Brackish or salty water contains salt ions that can be removed through a spacer channel with porous electrodes on either side. When a potential difference between the two electrodes is applied, cations (Na^+^) concentrate in the cathode while the anode absorbs anions (Cl). This process results in partially desalinated water. A concentrated salt product stream is discharged when the electrodes release ions over time, and the voltage is reduced or even reversed. Ion-exchange membranes must be placed between the electrodes and the spacer to reverse the voltage in the membrane-C variation of CDI [28,29].

### 2.1. Design and Fabrication of the CDI Desalination Unit

This study explores structural design guidelines for CDI setups that are simple to assemble, use, and maintain. The anode and cathode, two electrodes connected to a direct current power source, exhibit opposing positive and negative charges, respectively. The brackish or salty water from the tank was pumped into the CDI cell. The voltage was reversed to regenerate the electrodes after water deionization polluted both.

**Figure 1:**
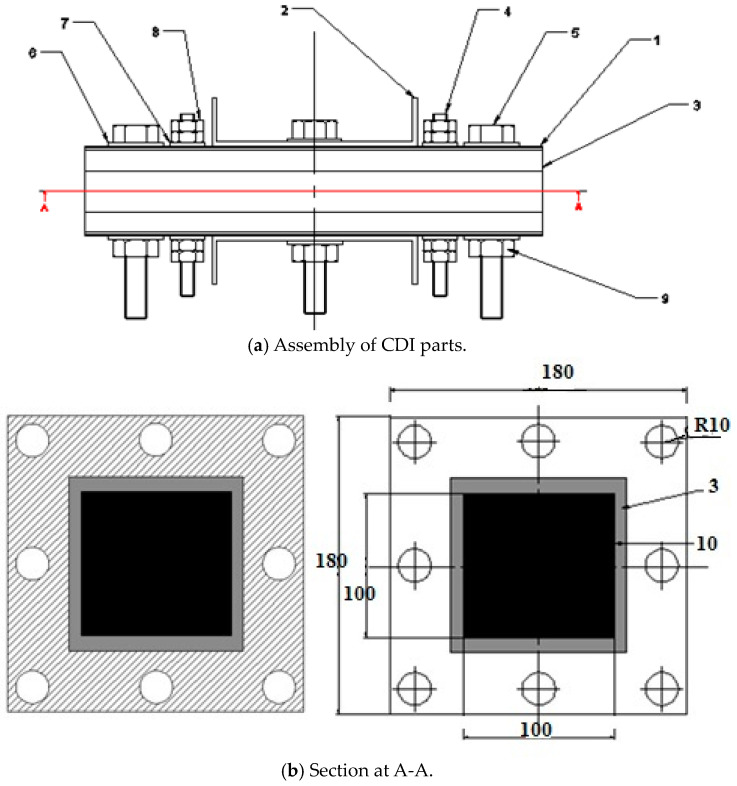
(**a**) Assembly of CDI parts. (**b**) Inside view section for CDI.

They were fabricated and assembled to design and manufacture the capacitive deionization (CDI) unit, illustrated in Figure 1 and Table 1.

### 2.2. Equipment Used in This Study

#### 2.2.1. DC Power Supply

A DC power source (XLN6024, made in Taiwan) is an electronic component that offers electrical power to an electronic device. The device acquires electrical energy from a power source. It converts the AC into DC, which is crucial for the CDI cell to enable the electrolytic separation of salts from water. Furthermore, it regulates the voltage across different levels to explore the influence of electric potential on cellular functionality.

#### 2.2.2. Peristaltic Pump

Peristaltic pumps (77800-60, made in Italy) are a type of positive displacement pump that employ the action of rotating rollers in contact with specialized flexible tubing to generate a flow under pressure. The tubing effectively segregates the fluid from the surrounding pump and its environment, thereby preventing contamination. This feature renders the tubing highly suitable for manipulating aggressive, corrosive, or abrasive substances. The roller mechanism also facilitates the low-shear pumping of fluids, particularly those sensitive to shear forces.

#### 2.2.3. Tubular Furnace

An electric heating apparatus (TUBE-1400, made in China) is employed to conduct synthesis and purify inorganic compounds and, occasionally, inorganic synthesis. A potential design configuration entails the utilization of a cylindrical cavity encompassed by heating coils integrated into a thermally insulating matrix. Temperature regulation can be achieved by using feedback obtained from a thermocouple. Tube furnaces with multiple heating zones are commonly employed in transport experiments. Specific digital temperature controllers offer an RS232 interface, enabling users to program segments for various purposes, such as ramping, soaking, sintering, and other applications.

#### 2.2.4. Digital Balance

The digital mass balances utilized in general chemistry laboratories (DAM, made in the UK) are exact instruments employed to measure the weight of substances up to the milligram (0.001 g) level. It is imperative to handle them with caution and attentiveness. Employing appropriate containers to weigh chemicals and ensure that objects are weighed under ambient room temperature conditions is advisable. It is advisable that the draft shields be maintained in a closed position. It is imperative that any disruption or alteration to the instruments or their respective levels be avoided. It is essential for cleanliness to be consistently maintained near the pan by employing a sable brush for cleaning after use. Additionally, it is necessary that the stockroom personnel be promptly notified of any spillage of liquids or solids onto the balance.

#### 2.2.5. PH/EC/TDS Meter

A TDS meter (MW 802, made in Romania) can measure the total soluble solids in a solution. Precisely, it measures the concentration of solid particles that are dissolved within it, focusing on the existence of ionized particles such as minerals and salts. The solution’s electrical conductivity (E.C.) is increased due to these dissolved particles. In a solution, dissolved particles increase the electrical conductivity (E.C.). This specific solution feature can calculate the total dissolved solids (TDS). The E.C. is a helpful tool because it can measure ionized solids.

#### 2.2.6. Vacuum Oven

The vacuum furnace (281A, made in the USA) is a specialized furnace utilized to subject materials, particularly metals, to elevated temperatures, thereby facilitating various processes, including, but not limited to, brazing, sintering, and heat treatment. This furnace ensures high uniformity and minimal contamination during these operations.

#### 2.2.7. Ice Generator

The ice generator (CIM24A60HZ, made in the UK) is the ice machine component responsible for ice production. This encompasses the evaporator and any related drives, controls, and subframe components that play a direct role in the production and discharge of ice into storage. The “ice generator” typically refers to the ice-making subsystem in isolation, excluding refrigeration components. An ‘exceptionally packaged ice machine’ generally refers to a fully integrated apparatus encompassing refrigeration and control systems, only necessitating connection to utility services.

#### 2.2.8. Stirrer

A magnetic stirrer (Stuart SB162, made in the UK), also known as a magnetic mixer, is a laboratory instrument that utilizes the rotation of a magnetic field to induce the rapid spinning of a stir bar, commonly referred to as a “flea”, which is submerged in a liquid. This spinning motion effectively stirs the liquid. A rotating magnet or a collection of stationary electromagnets positioned beneath the liquid-filled vessel can generate a rotating field.

#### 2.2.9. Grinder

Grinders (IP15AP, made in the UK) are deemed appropriate for achieving consistent sample preparation, essential for subsequent analysis within the context of quality assurance and Good Laboratory Practice (GLP). Additionally, grinders are also utilized in the process of preparing prescriptions and formulations. Challenging-to-comminute samples can be effectively processed in grinders through thermal manipulation, either by heating or cooling or by incorporating grinding aids.

### 2.3. Carbonization Process

The capacitive deionization electrode was constructed using natural materials in this study. The term “high pours” refers to the pouring of a substantial amount of liquid, typically a beverage. Water desalination was conducted utilizing carbon-activated nanomaterial or graphene oxide. In the present investigation, various natural materials such as rice, peanut, and sunflower husk were employed to create carbon-activated substances. In addition to sunflower seeds, peanut shells, and rice husks, various other materials can be processed to produce activated carbon through carbonization:

- Coconut shells, known for their high carbon content and low ash content, making them ideal for activated carbon production;
- Wood chips and sawdust, commonly used due to their availability and ability to produce high-quality activated carbon;
- Fruit pits, such as olive and cherry pits, which are rich in carbon and well suited for activation;
- Agricultural residues like corn stover, sugarcane bagasse, and wheat straw, which are abundant and often underutilized;
- Bone char, made from animal bones, offering a unique composition for specific adsorption applications.

**Figure 2:**
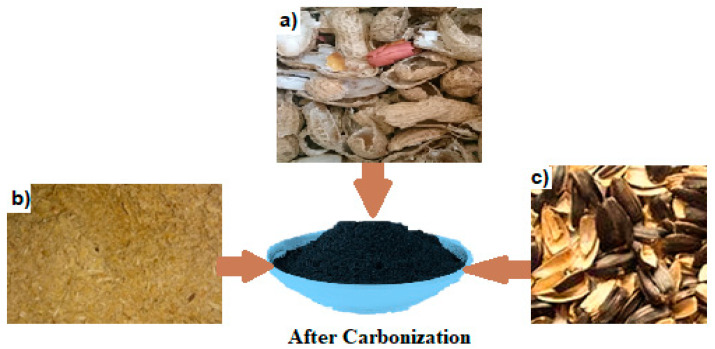
Nature materials before carbonization and after carbonization: (**a**) peanut peels, (**b**) rice peels, and (**c**) sunflower peels.

Subsequently, these substances were utilized to construct CDI electrodes, which were integrated into the configuration of CDI desalination systems, as shown in the experimental procedure outlined in Figure 2.

The shells of sunflower seeds, peanuts, and grains were all finely ground. The product was dried at 200 °C for 19 h. The final step involved grinding the material and loading it into quartz boats. Carbonization finally occurred after heating the furnace to 700 °C and maintaining it at this temperature for an hour with a constant flow of 18 dm^3^/min of argon. After undergoing pyrolysis, the samples were again milled down to ground level. The resulting pH was neutral when the powder was washed with distilled water. After soaking in 1 mol/dm^3^ concentrated hydrochloric acid for 19 h, the following procedures were carried out to neutralize the filtrate.

### 2.4. Chemical Activation

**Figure 3:**
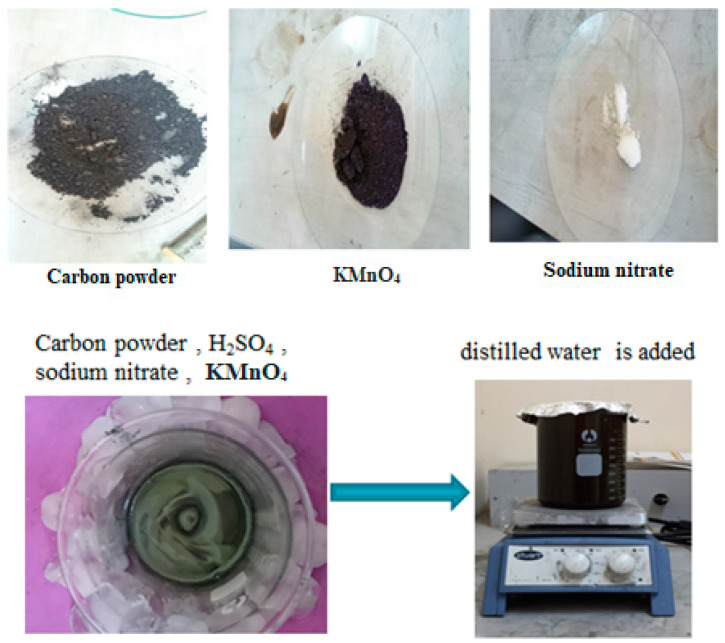
The procedures and steps for activating chemical carbon.

Five grams of carbon powder were combined with 140 mL of concentrated H_2_SO_4_, 15 g of sodium nitrate, and 3 g of potassium permanganate, which were added gradually while stirring for 90 min. Slowly, 230 mL of distilled water was incorporated, as illustrated in Figure 3.

The A.C. or graphene oxide obtained was filtered, washed to remove all traces of acid, and then dried under vacuum. During the activation process with potassium hydroxide, activated carbon with potassium hydroxide is oxidized in an alkaline medium with a large amount of oxygen [26,30]. Carbon atoms are removed from the internal structure of carbon during vigorous activation with a large amount of potassium hydroxide, increasing the surface area [31].

### 2.5. Electrode Sheet Fabrication

**Figure 4:**
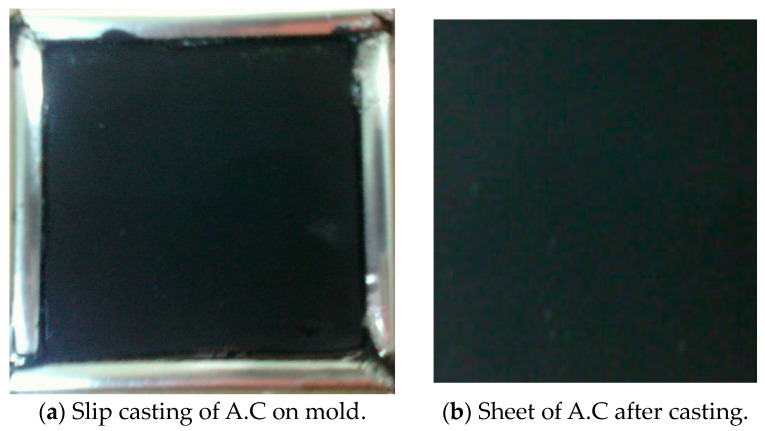
(**a**,**b**) Preparation of electrode sheets by slip casting onto 10 cm × 10 cm mold.

**Figure 5:**
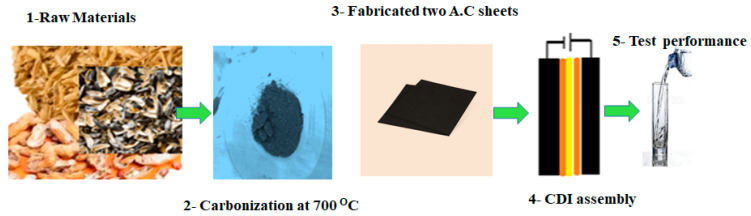
Steps of the synthesis of an electrode sheet from raw materials.

Activated carbon, a binder, and a conductive additive are added to the current collector before the electrode is produced by slip casting. Attaching the ACP to the current collector and giving it structural rigidity is the job of the polymeric (PVDF) binder. Binders, such as polyvinylidene fluoride (PVDF), are widely employed [32]. At 150 °C, activated carbon powder, ethyl acetate, and polyvinylidene fluoride (PVDF) are mixed and agitated. Since excessive binder could diminish the surface area by blocking access to pores, using the tiniest quantity necessary to give the electrode appropriate mechanical strength is standard practice. About 10% by weight (wt%) comprised PVDF [33]. Conductive binders and ACP are slurried together in a suitable solvent to form a slurry. The Figure shows that the casting or coating procedure in the mold dimension can affect the CDI electrode’s characteristics and properties. After casting, the electrode is dried at a high temperature, often in a vacuum, until the solvent completely evaporates [34]. We utilize carbon sheet drying at 120 °C for 2 h and vacuum oven drying at 80 °C for 2 h. For 24 h, we treat the product with a 1 M KOH solution, followed by drying at 70 °C in a vacuum for 6 h after being washed with deionized water until the pH is neutral [35], as shown in Figure 4 and Figure 5.

### 2.6. Description of the Assembly Electrodes of the CDI Cell

**Figure 6:**
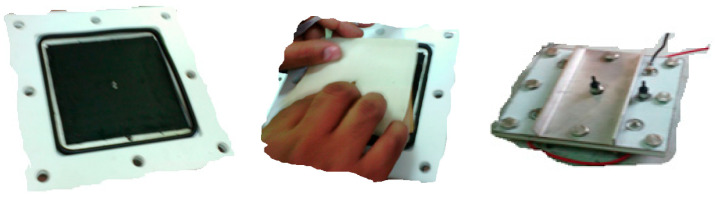
Assembly of two electrode sheets and themembrane inside the CDI cell.

Figure 6 shows the CDI unit cell. Carbon electrodes are produced with the dimensions 10 × 10 cm^2^ and are used as the anodes and cathodes. The capacitive deionization method has been modified by introducing membrane capacitive deionization (MCDI), whereby ion-exchange membranes are strategically placed in front of every carbon electrode. MCDI is reported to significantly improve desalination efficiency [36], because ions are selectively adsorbed onto the electrode surface by an ion-exchange membrane. The anode was formed by combining the anion-exchange membrane (AMX) with the carbon electrode. At the same time, the cathode was created by combining the carbon electrode with the cation-exchange membrane (CMX) [37]. A 20 µm thick spacer (a nylon net) was inserted between the ion-exchange membranes to form a flow channel for the influent. A 1 cm hole was drilled in the center of the carbon electrode, which served as the outlet. The device was designed so that influent flows in from the edges of the carbon electrode, passes through the spacer, and then flows out through the drill hole in the center of the electrode.

### 2.7. Desalination Processes Using CDI Unit

**Figure 7:**
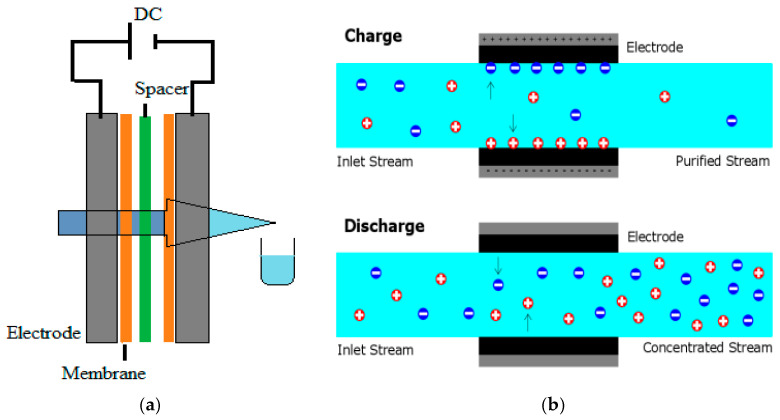
Schematic diagram of desalination process by CDI: (**a**) general processes and (**b**) charge and recharge.

A series of desalination processes was conducted using a CDI unit cell to evaluate the efficiency of CDI design performance, as illustrated in Figure 7. Various parameters were applied to measure the effectiveness of the CDI design.

For the desalination of brackish water with TDS concentrations between 200 and 5000 ppm, capacitive deionization (CDI), a developing method, has shown potential to be cost-effective [38]. A direct current (DC) voltage is supplied to a cell made up of a pair of electrodes in CDI, giving the technology its name. Ions are adsorbed onto the charged electrode surfaces, while saline water is circulated through the cell through an insulating spacer to prevent short circuits. This procedure is known as electrosorption, since the applied voltage acts as the driving force for adsorption.

The applied voltage can be reduced or changed in polarity to cause the ions to be resorbed once the electrode surfaces are saturated with ions. Then, purified water and concentrated brine can be produced by switching between the adsorption (charge) and desorption (discharge) stages [39].

## 3. Desalination Experimental Procedures

Traditional CDI systems consist of two steps: a charge step, in which a voltage is supplied and ions are adsorbed in the cell, and a discharge step, in which the voltage changes in polarity or is shorted to eliminate the charged particles from the saturation electrodes. The circulation of saltwater through the CDI cell causes this to occur. Various parameters, such as flow velocity, temperature, applied voltage, and salt concentrations, influence the operation of conventional CDI systems.

According to the water treatment application, these operating parameters that impact the CDI system can occasionally be adjusted to obtain the required desalination performance or reduce the cost of water production. New operating modes, such as constant current rather than constant voltage, have also been developed to make CDI a more varied desalination technique.

The desalination process depends on many variables; we selected the concentration of TDS, flow rate of feed, and volt of electrodes in CDI.

The following parameters were studied:

1. The saline concentration of seawater TDS was measured at 500, 1000, 1500, and 2000 TDS.
2. The flow rate of feed was 9.5, 16.8, and 38.2 mL/min.
3. The potential difference in the electrode was calculated at 1.5, 2 and 2.5 volts for an interval of 1 min.

## 4. Results and Discussion

Traditional CDI systems consist of two steps: a charge step, in which a voltage is supplied and ions are adsorbed in the cell, and a discharge step, in which the voltage changes in polarity or is shorted to eliminate the charged particles from the saturation electrodes. Various parameters, such as flow velocity, temperature, applied voltage, and salt concentrations, influence the operation of conventional CDI systems.

According to the water treatment application, these operating parameters that impact the CDI system can occasionally be adjusted to obtain the required desalination performance or reduce the cost of water production. New operating modes and techniques, such as employing constant current instead of constant voltage, have been developed to enhance the versatility of CDI as a desalination method [40,41].

### 4.1. Effect of the Concentration of TDS on Desalination

CDI technology is frequently tested in brackish water salinities below 2000 ppm TDS. At these salinities, CDI technology is effective. According to adsorption isotherm models, the salt adsorption capacity and the influent salt concentration should increase [42]. The charge efficacy decreases as the NaCl content increases, proving that low-salinity water is best for CDI technologies. However, research has shown that the effect of NaCl content on charge efficacy is minimal, if not insignificant. The EDLC hypothesis predicts that higher salt concentrations will cause more ions to pass through the electrodes and a higher capacitance. A higher influent NaCl concentration significantly enhances the energy consumption and electrosorption kinetics of the desalination process, because the water is more conductive [43].

The ion species is particularly significant, since salts other than NaCl are frequently present in source water matrices.

**Figure 8:**
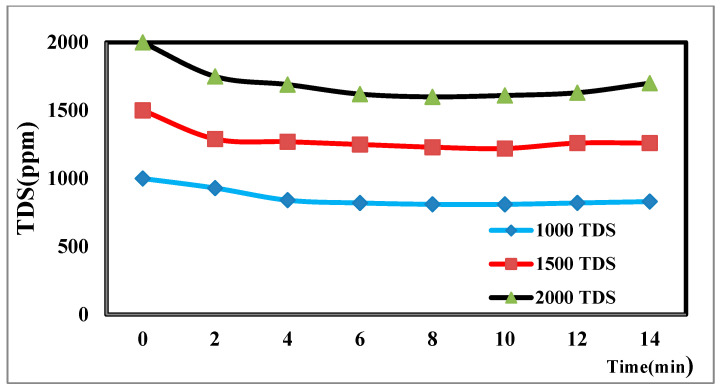
Effect of different concentrations on desalination.

**Figure 9:**
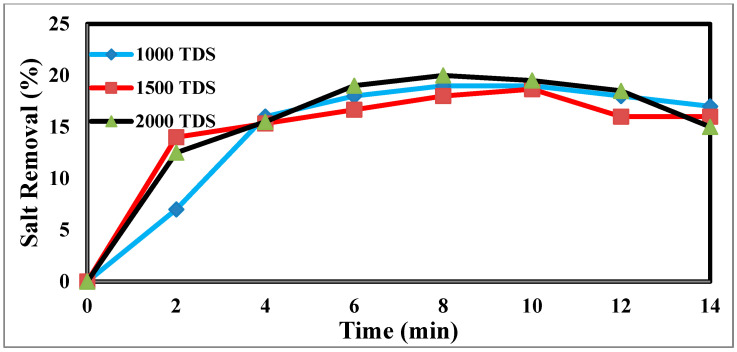
The relation between the removal % and time for ions.

The TDS concentration affects the desalination process via CDI [44]. The different TDS concentrations in Figure 8 illustrate how the TDS ion concentration changes over time. The result shows that TDS increases at a flow rate of 25 mL/min, has a potential difference of 1.5 volts, and decreases from 2000 ppm to 1610 ppm after the anode and cathode have stabilized. The same rules apply for 1000 p.p.m. and 1500 p.p.m. with constant flow rates and different potentials. The relationship between the removal percentage and time for ions is shown in Figure 9.

### 4.2. Effect of Flow Rate Feed on Desalination

**Figure 10:**
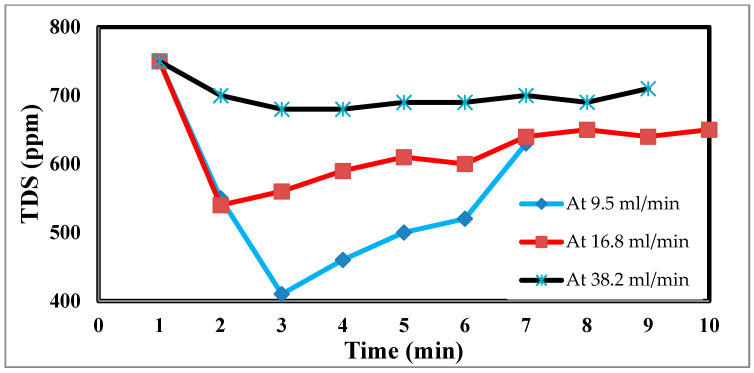
Effect of different flow rates on desalination.

**Figure 11:**
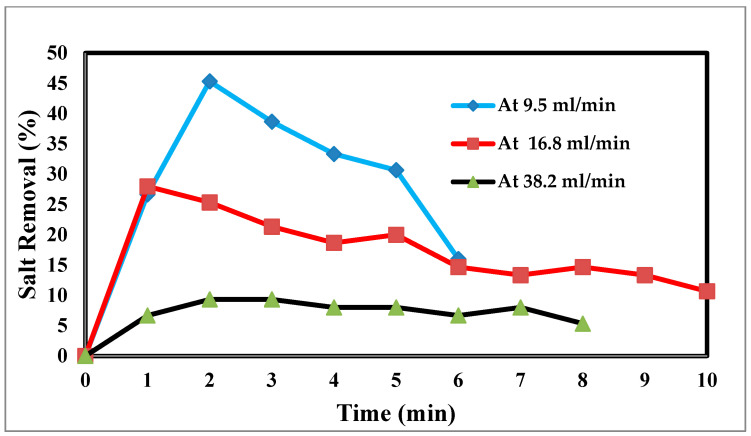
Relation between the removal % and time for ions and flow rate.

In an experimental setting, the flow rate is crucial, because it regulates how long the salt stays in contact with the electrodes. The effluent concentration is more filtered due to the saline water’s lengthier contact with the electrodes and increased salt removal effectiveness at low flow rates [45]. The relationship between the flow rate and energy consumption is inverse, although the flow rate also has positive relationships with the charge efficiency and salt adsorption capacity, as well as with both [46]. For the most efficient use of energy and salt removal, it is recommended that a pass CDI system with two sheets of activated carbon electrodes and electrode dimensions of 10 × 10 × 1 mm employ flow rates of 9.5, 16.8, and 38.2 mL/min. However, this optimal flow rate is 9.5 mL/min because of flow rate changes. Retention time, or the time that the saline water is in contact with the electrode, has thus been advocated as a potential operating factor in place of the flow rate. The flow rate input through CDI has an impact on the desalination process. The relationship between the feed flow rate velocity and feed TDS decrease is shown in Figure 10 at 9.5 mL/min, corresponding to a sharp TDS decrease. This demonstrates the direct correlation when 38.2 mL/min exhibits a tiny reduction, which informs us that a low flow rate allows time for ions to be drawn to the anode and cathode. The relationship between the removal percentage and time for ions is shown in Figure 10 and Figure 11.

### 4.3. Effect of Potential Differences on Desalination

**Figure 12:**
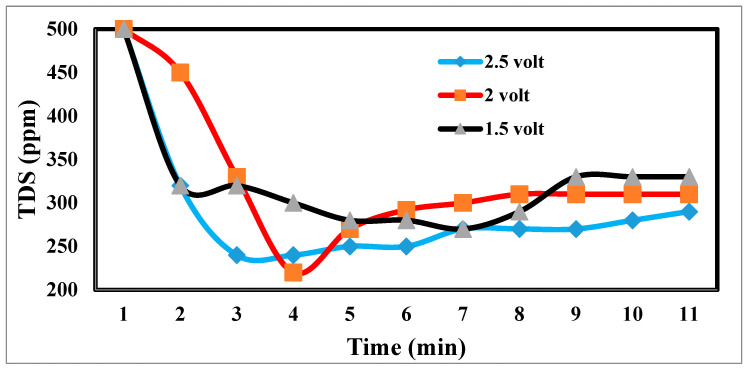
Effects of different potential differences on desalination.

**Figure 13:**
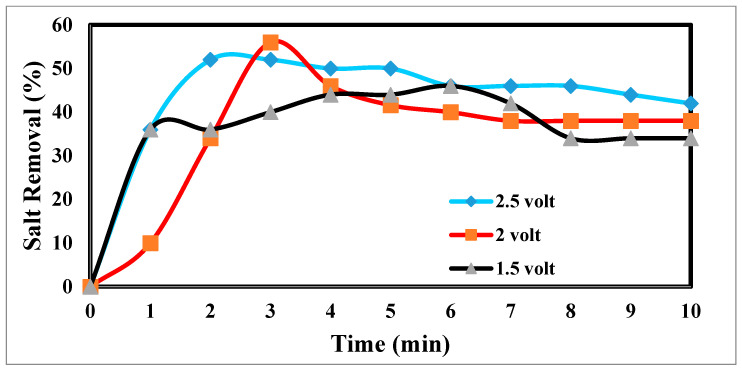
Relation between the removal and time for ions.

The desalination process is affected by potential electrode differences in CDI [47,48], as shown in Figure 12. It shows a sharp decrease in TDS at 2.5 and 2 volts, which appears to be the increase in volts that reduces TDS so fast [49]. Figure 13 indicates the relation between the removal % and time for ions. According to EDL theory, increasing the voltage applied to the CDI cell boosts the charge at the electrode–solution interface, promoting the salt adsorption capacity. The capacity for absorbing salt rises exponentially from 0.6 V to 1.2 V rather than linearly with applied voltage. The salt adsorption capacity, however, does not appreciably increase above 1.2 V, most likely because of parasitic electrochemical processes [50]. The standard reduction potential (SRP) for water electrolysis being at 1.23 V and data demonstrating that charge efficiency declines and specific energy consumption rise with increasing applied voltage support this. Different applied voltages have been reported to improve various desalination parameters, including salt adsorption capacity, charge efficiency, specific energy consumption, and electrosorption rate. Based on their findings, the authors of [51] asserted that 1.2 V, which is only marginally lower than the SRP for water electrolysis, is the ideal point.

On the other hand, they used the response surface approach in their study and discovered that 1.57 V had the highest salt adsorption capacity [44]. More experiments revealed that 2.0 V produced the lowest specific energy usage, and 1.8 V produced the fastest electrosorption rate. For most CDI systems, an applied voltage of 1.0 to 2.0 V is suitable [52]. The performance with the application of 2 volts is more efficient at 55%.

### 4.4. Regeneration of CDI Electrode

**Figure 14:**
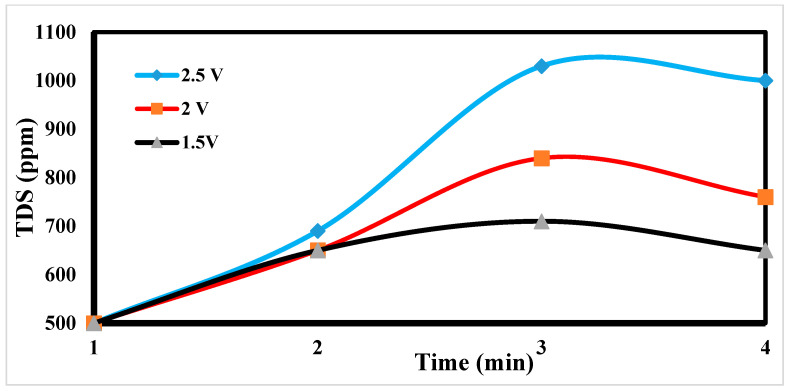
Regeneration of CDI electrode.

When we observe an increase in TDS, we change the electrode by changing the anode to the cathode and vice versa, which leads to the repulsion of ions deposited on the electrode. As Figure 14 shows a sharp and sudden increase in TDS for a while after that, we notice a slow decrease in grading; this shows the readiness of the electrode for a new desalination process. We also note that there is a relationship between low voltage [53] and TDS low voltage, giving us good regeneration.

## 5. Conclusions

There are various sources of carbon black from natural agricultural materials, such as rice husks, peanut shells, and sunflower husks. CDI desalination technology is relatively inexpensive compared to other methods. Several factors influence water desalination, including the electrode material, total dissolved solids (TDS), flow rate, and voltage. The average efficiency at a constant TDS of 750 ppm, voltage of 1.5 V, and varying flow rates of 9.5, 16.8, and 38.2 mL/min. The max. efficiency at 9.5 L/min is 45% at a time of 2 min. The best efficiencies at voltages of 2.5 V, 2 V, and 1.5 V after 3 min are attained with a consistent flow rate of 9.5 mL/min, 2 volts, and a TDS of 500 ppm. The maximum performance for desalination reaches 55%. We measured the effectiveness of desalination at varying TDS concentrations of 2000, 1500, and 1000, maintained at a constant flow rate of 25 L/min and 2 V. The peak efficiency achieved at the 8 min mark is between 18% and 20%.

## 6. Future Work

Future research should focus on optimizing the use of rice, peanut, and sunflower husks, as well as other agricultural waste, as sustainable sources of carbon black for applications such as activated carbon, graphene oxide, and carbon nanotube electrode sheets in CDI desalination technology. Testing these electrodes and materials is crucial in improving system performance. Ultimately, laboratory findings could inform smaller-scale experiments. Additionally, the economic viability of CDI technology utilizing eco-friendly materials across various settings for sustainable water management will be assessed.

## Figures and Tables

**Table 1:** Descriptions of assembly parts of CDI unit.

Part No.	Name of Part	Type of Material	Qty.
1	Stainless steel plate	Stainless steel	2
2	Stainless steel cover (U-shape)	Stainless steel	2
3	Tofflen polymer plate	Tofflen (polymer)	4
4	Bolt without head	Stainless steel	6
5	Bolt with head	Stainless steel	6
6	Washer	Stainless steel	12
7	Washer	Stainless steel	8
8	Hex. nut	Stainless steel	12
9	Hex. nut	Stainless steel	6
10	Activated sheet	Activated carbon nanoparticles	1

## Data Availability

The original contributions presented in the study are included in the article. Further inquiries may be directed to the corresponding author (due to privacy).
